# Marizomib sensitizes primary glioma cells to apoptosis induced by a latest-generation TRAIL receptor agonist

**DOI:** 10.1038/s41419-021-03927-x

**Published:** 2021-06-24

**Authors:** Chiara Boccellato, Emily Kolbe, Nathalie Peters, Viktorija Juric, Gavin Fullstone, Maïté Verreault, Ahmed Idbaih, Martine L. M. Lamfers, Brona M. Murphy, Markus Rehm

**Affiliations:** 1 Institute of Cell Biology and Immunology, University of Stuttgart, Stuttgart, Germany; 2 Department of Physiology and Medical Physics, Royal College of Surgeons in Ireland, Dublin, D2 Ireland; 3 Stuttgart Research Center Systems Biology, University of Stuttgart, Stuttgart, Germany; 4 Inserm U 1127, CNRS UMR 7225, Sorbonne Universités, UPMC Univ Paris 04 UMR S 1127, Institut du Cerveau, ICM, Paris, France; 5 Sorbonne Université, Inserm, CNRS, UMR S 1127, Institut du Cerveau de Paris, ICM, AP-HP, Hôpitaux Universitaires La Pitié Salpêtrière-Charles Foix, Service de Neurologie 2-Mazarin, Paris, France; 6 Department of Neurosurgery, Brain Tumor Center, Erasmus MC, Rotterdam, The Netherlands

**Keywords:** CNS cancer, Cell biology

## Abstract

Due to the absence of curative treatments for glioblastoma (GBM), we assessed the efficacy of single and combination treatments with a translationally relevant 2nd generation TRAIL-receptor agonist (IZI1551) and the blood–brain barrier (BBB) permeant proteasome inhibitor marizomib in a panel of patient-derived glioblastoma cell lines. These cells were cultured using protocols that maintain the characteristics of primary tumor cells. IZI1551+marizomib combination treatments synergistically induced apoptotic cell death in the majority of cases, both in 2D, as well as in 3D spheroid cultures. In contrast, single-drug treatments largely failed to induce noticeable amounts of cell death. Kinetic analyses suggested that time-shifted drug exposure might further increase responsiveness, with marizomib pre-treatments indeed strongly enhancing cell death. Cell death responses upon the addition of IZI1551 could also be observed in GBM cells that were kept in a medium collected from the basolateral side of a human hCMEC/D3 BBB model that had been exposed to marizomib. Interestingly, the subset of GBM cell lines resistant to IZI1551+marizomib treatments expressed lower surface amounts of TRAIL death receptors, substantially lower amounts of procaspase-8, and increased amounts of cFLIP, suggesting that apoptosis initiation was likely too weak to initiate downstream apoptosis execution. Indeed, experiments in which the mitochondrial apoptosis threshold was lowered by antagonizing Mcl-1 re-established sensitivity to IZI1551+marizomib in otherwise resistant cells. Overall, our study demonstrates a high efficacy of combination treatments with a latest-generation TRAIL receptor agonist and the BBB permeant proteasome inhibitor marizomib in relevant GBM cell models, as well as strategies to further enhance responsiveness and to sensitize subgroups of otherwise resistant GBM cases.

## Introduction

Glioblastoma (GBM) is the most aggressive cancer of the central nervous system. Surgical resection, adjuvant temozolomide-based chemotherapy, and radiation are the primary treatments, yet the outcome of GBM patients remains poor with a median life expectancy of 15-17 months [1–3]. Novel and effective treatment options are therefore required, as are reliable pre-clinical experimental models that are suitable for exploratory studies on novel drugs and drug combinations.

In recent years, it has been understood that conventional GBM cell cultures that have traditionally been used to study drug responsiveness, associated signaling, and cell death outcomes poorly reflect the transcriptome characteristics of the original tumors. Advanced culturing conditions and limited cultivation times are now becoming more widely accepted as the state-of-the-art to maintain superior cell line models that closely resemble original tumor characteristics [4–6]. Such models were applied in this study, in which we investigated the responsiveness of GBM cells to combinations of marizomib and a latest-generation TRAIL receptor agonist (IZI1551) [7, 8].

Marizomib was originally isolated from sedimentous marine bacteria as a highly potent inhibitor of the chymotrypsin-like proteolytic activity of the 20S proteasome [7]. Compared to therapeutically approved proteasome inhibitors such as bortezomib and carfilzomib, marizomib inhibits the proteasome similarly effectively and additionally can cross the blood–brain barrier [9–11]. Consequently, various clinical trials in which marizomib is tested as a single agent or in combination treatments, including in glioblastoma, have been initiated [9] (NCT03345095). However, it remains to be seen if marizomib improves GBM patient outcome from radio-chemotherapy.

Prolonged proteasome inhibition induces proteotoxic stress that ultimately results in apoptotic cell death. The stress response and apoptosis induction upon proteasome inhibition are complex and, among other processes, includes the accumulation or stabilization of otherwise short-lived pro-apoptotic proteins, the induction of the unfolded protein response and active induction of intrinsic apoptosis, as well as the formation of platforms on which the apoptotic caspase-8 can be activated, a protease otherwise implicated in extrinsic death receptor-induced apoptosis [12, 13]. Proteasome inhibition and death receptor activation can synergistically induce apoptosis, as demonstrated already 25 years ago [14]. Synergies likely arise from the complex interplay of multiple cellular responses. These include, for example, the suppression of pro-survival signaling by IκBα stabilization, the accumulation of death receptors, the stabilization of activated caspases, and the convergence of extrinsic and intrinsic apoptosis signaling branches at the level of mitochondria, resulting in the permeabilization of the outer membrane of the latter [13, 15–17]. Increased or synergistic apoptosis induction arising from the combination of proteasome inhibitors and death ligands have been described in conventional GBM cells line models, such as U87MG and T98G cells treated with the proteasome inhibitor MG132 or bortezomib and recombinant human TRAIL [18, 19]. This has also been observed in primary GBM cells and GBM stem cells cultured in the serum-free medium when treated with bortezomib and recombinant human TRAIL [20]. Substantial progress has been made in the development of translationally relevant TRAIL receptor agonists, with highly potent hexavalent formats having been reported that entered clinical trials [8, 21, 22]. Among these new variants is IZI1551, a highly stable hexavalent format that in TRAIL sensitive cells induces apoptosis at LD50 concentrations in the pM to low nM range [8, 23]. While these biologics would be expected to be too large to cross the blood–brain barrier during systemic therapy, implantation of wafers or polymers that release these agents might offer an avenue for clinical application in the GBM setting in the future.

Here, we studied the efficacy of combination treatments of marizomib and IZI1551 in patient-derived cell line models generated from fresh primary or recurrent GBM tumors to assess the prevalence of responsiveness in 2D and 3D culture conditions and to identify sensitization strategies where marizomib/IZI1551 combinations remain ineffective.

## Results

### The majority of patient-derived glioblastoma cell lines respond to combination treatment with a 2nd generation TRAIL receptor agonist and marizomib

**Fig. 1:**
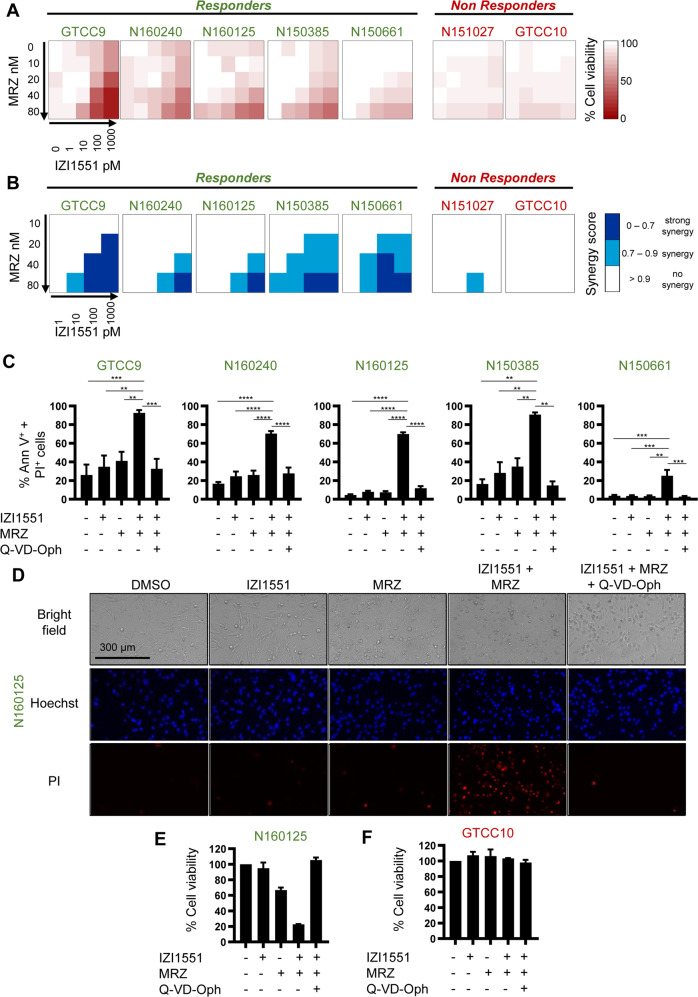
Most patient-derived glioblastoma cells respond to combination treatment with a 2nd generation TRAIL receptor agonist and marizomib. **A** Cells were cultivated in 2D and stimulated with the indicated concentrations of IZI1551 and marizomib for 24 h. Cell viability was assessed by WST-1 cell proliferation assay. Data are mean values from three independent experiments. SEMs across the repeat experiments and conditions were <20%. **B** Synergy scores of drug combinations were determined by calculating Webb’s fractional product. **C** Glioblastoma cells were treated for 24 h with IZI1551 (1 nM), marizomib (80 nM), or with the combination thereof in the presence or absence of the pan-caspase inhibitor Q-VD-Oph (50 μM). Cell death was measured by Annexin V/PI-based flow cytometry. Data represent mean ± SEM from three independent experiments. **p ≤ 0.01; ***p ≤ 0.001 ****p ≤ 0.0001; one-way ANOVA followed by Tukey post hoc test. **D** Representative micrographs of N160125 cells after 24 h of treatment as in **C**. Nuclei were stained with Hoechst and PI. Nuclear condensation and membrane blebbing indicate apoptotic cell death. **E**, **F** Long-term proliferation capacity was tested in responder and non-responder cell lines treated for 24 h as in **C**. Viability signals of survivor populations were measured after 6 days of recovery by WST-1 assays. Bar graphs show the mean ± range of two independent experiments per cell line.

To identify if glioblastoma cells can respond to a representative 2nd generation TRAIL receptor agonist (IZI1551), alone or in combination with proteasome inhibitor marizomib, we used primary cell cultures isolated from seven patient tumors and established low passage cell lines following best practice protocols [4, 5]. For 25 conditions per cell line, cell viability was analyzed after 24 h of treatment by WST-1 assays (Fig. 1A). While responsiveness to the individual drugs was generally poor, combination treatments substantially lowered cell viability in the majority of cell lines (Fig. 1A, responders). Moreover, the drug interactions evoked robust synergies in responsive cell lines, as identified by calculating Webb’s fractional products [24] (Fig. 1B). Of note, loss in viability correlated with induction of cell death when determining the latter by annexin V/propidium iodide (AV/PI) flow cytometry (Supplemental Fig. 1). The addition of the pan-caspase inhibitor Q-VD-Oph reduced cell death to background levels, indicating that cell death execution was caspase-dependent in this treatment scenario (Fig. 1C; note that a number of cell lines, in particular GTCC9 cells, appeared highly sensitive to detachment and mechanical stress arising from liquid handling, reproducibly resulting in notable background cell death at control conditions in flow cytometry experiments). Co-staining cells with Hoechst 33258 and PI likewise confirmed substantially increased amounts of cell death for IZI1551+marizomib co-treatment conditions (Fig. 1D). Cellular and nuclear morphologies, such as rounding/blebbing and karyopyknosis, and their absence in presence of Q-VD-Oph, indicated that cell death occurred by apoptosis (Fig. 1D).

Besides acute induction of caspase-dependent cell death, IZI1551+marizomib treatment might additionally impair the continued proliferation of surviving fractions of cells. To study long-term proliferation capacity, we re-plated identical numbers of surviving cells after 24 h of treatment and measured cell viabilities after six days. While marizomib treatment reduced the long-term proliferation capacity of surviving cells by approximately 30%, combination treatment with IZI1551+marizomib potently prevented the proliferation of surviving cells (Fig. 1E). Of note, caspase inhibition completely re-established proliferation capacity in the combination treatment setting (Fig. 1E). In contrast, none of the treatments affected long-term proliferation capacity in the non-responder cell line GTCC10 (Fig. 1F).

Overall, these findings demonstrate that the majority of freshly established GBM cell lines synergistically respond to the combination of the proteasome inhibitor marizomib and a latest-generation TRAIL receptor agonist, with the activation of caspases being required for cell death execution and to minimize long-term proliferation capacity.

### IZI1551+marizomib co-treatments are effective in 3D tumor cell spheroid models

Two-dimensional cell cultures might oversimplify the complexity of cell-to-cell interactions, with the inherent danger of underestimating drug resistance mechanisms that potentially can manifest from 3D microenvironments [25, 26]. Therefore, we validated IZI1551+marizomib treatment efficacy also in 3D tumor cell spheroids.

**Fig. 2:**
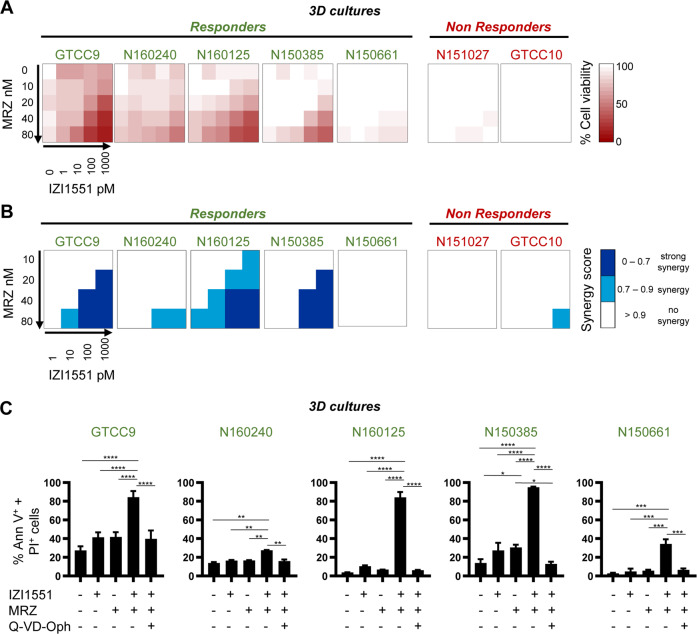
IZI1551+marizomib co-treatments are effective in 3D tumor cell spheroid models. **A** Cell lines were cultivated as a 3D spheroid and stimulated with the indicated concentrations of IZI1551 and marizomib for 24 h. Cell viability was assessed by WST-1 assays. Data are mean values from three independent experiments. SEMs across the repeat experiments and conditions were <20%. **B** Synergy scores of drug combinations were determined by calculating Webb’s fractional product. **C** Spheroids were treated with IZI1551 (1 nM) or marizomib (80 nM) or a combination of both in the presence or absence of the pan-caspase inhibitor Q-VD-Oph (50 μM). Cell death was measured by Annexin V/PI-based flow cytometry. Data represent mean ± SEM from three independent experiments. *p ≤ 0.05; **p ≤ 0.01; ***p ≤ 0.001 ****p ≤ 0.0001; one-way ANOVA followed by Tukey post hoc test.

Based on viability loss measurements, the cell line panel could still be separated into responsive and resistant cell lines, with only small changes in their sensitivity to the treatments in 3D versus 2D conditions (Fig. 2A). Controls conducted by microscopic imaging confirmed cellular viability loss and disintegration of spheroids upon combined treatment with marizomib and IZI1551 (Supplemental Fig. 1B). Correspondingly, synergy scores for the combination treatments were mostly maintained in the 3D setting, with the exception of N150661 cells (Fig. 2B). Furthermore, IZI1551+marizomib treatment-induced cell death remained caspase-dependent also in spheroid cultures, as assessed via flow cytometry measurements (Fig. 2C). Overall, these data demonstrate that results obtained in conventional 2D cell cultures are also reproducible in the more complex 3D growth scenario.

### Marizomib pre-treatment accelerates and enhances IZI1551-induced cell death

**Fig. 3:**
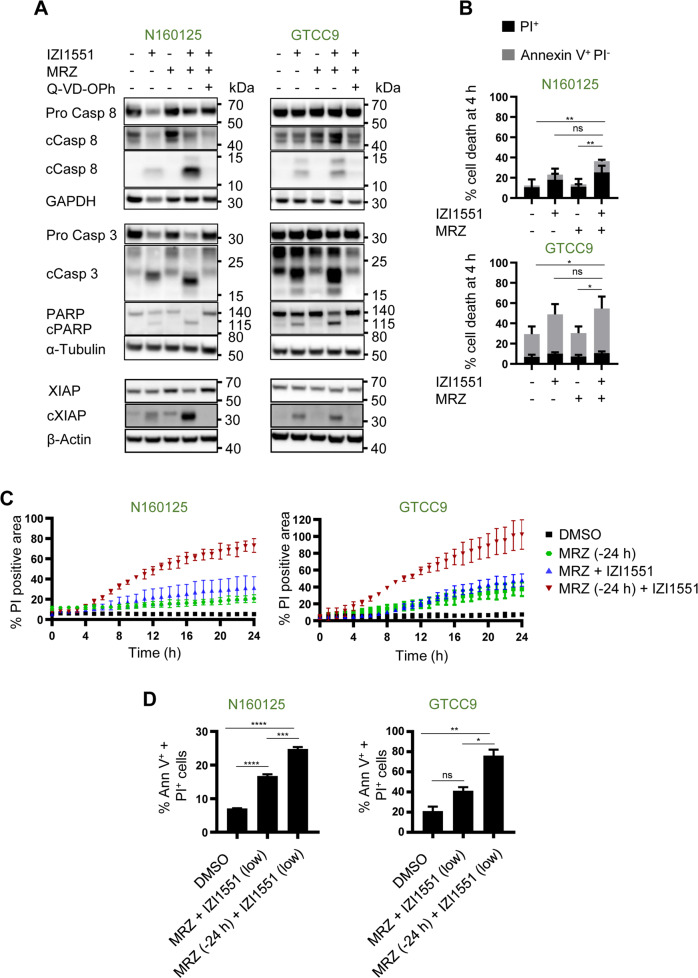
Marizomib pre-treatment accelerates and enhances IZI1551-induced cell death. **A** Caspase processing and caspase substrate cleavage in responder cell lines at early times. Cells were treated for 4 h with IZI1551 (1 nM) or marizomib (80 nM) or a combination of both in the presence or absence of Q-VD-Oph (50 µM). Whole-cell lysates were analyzed for the indicated proteins by western blotting. GAPDH, α-Tubulin, or β-Actin served as loading controls. Similar results were obtained in independent repeat experiments. c, cleaved. **B** Early cell death responses measured by Annexin V/PI-based flow cytometry. Cells were treated as in **A**. Data represent mean ± SEM from three independent experiments. *p ≤ 0.05; **p ≤ 0.01. ns = non-significant; one-way ANOVA followed by Tukey post hoc test. **C** Quantification of cell death kinetics, calculated as a percentage of PI-positive cell areas. Cells were co-treated with IZI1551 (1 nM) and marizomib (80 nM) simultaneously or pre-treated with marizomib for 24 h (MRZ −24 h) before the addition of IZI1551. Representative results from one out of 3 independent experiments are shown. Error bars represent the SD of 3 technical replicates. **D** Annexin V/PI-based flow cytometry of cells co-treated with reduced concentrations of IZI1551 (100 pM) and marizomib (40 nM). Data represent mean ± SEM from three independent experiments. *p ≤ 0.05; **p ≤ 0.01; ***p ≤ 0.001; ****p ≤ 0.0001; ns = not significant; one-way ANOVA followed by Tukey post hoc test.

We next sought to identify if altering the relative timing of drug exposure can enhance treatment responsiveness and allow lowering drug concentrations without losing treatment efficacy. The rationale for this was that TRAIL receptor activation is known to trigger rather swift apoptosis [27, 28], whereas proteasome inhibition triggers complex cellular responses and requires longer times to induce cell death [29, 30]. We therefore first performed experiments with the intention to validate if initiation of cell death signaling kinetically differs between IZI1551 and marizomib treatments. At early times (4 h), responder cell lines N160125 and GTCC9 only incompletely activated the initiator caspase-8 and effector caspase-3, resulting in cleavage of the canonical caspase-3 substrate PARP, after single-agent IZI1551 and IZI1551+marizomib combination treatment, but not after exposure to marizomib alone (Fig. 3A). Cleavage of caspase-3 and PARP already at early times appeared more efficient upon combination treatment. This was also reflected in the cleavage of XIAP, the most potent intracellular inhibitor of caspase-3 but also a caspase-3 substrate [31] (Fig. 3A). Caspase processing and substrate cleavage were expectedly suppressed in presence of caspase inhibitor Q-VD-Oph. Correspondingly, IZI1551 treatments and IZI1551+marizomib combination treatments induced similar amounts of apoptosis at early times, with portions of cells exposing phosphatidylserine but not yet taking up PI (Fig. 3B). Likewise, only a few if any cells were identified as PI-positive at early times when observing adherent cell populations by fluorescence microscopy (Supplemental Fig. 2A). These findings support that IZI1551-induced apoptosis responses manifest earlier than marizomib-induced cell death responses and also that synergistic induction of apoptosis signaling requires prolonged combination treatment.

From these results, we postulated that pre-treatment with marizomib might allow an enhancement of treatment synergies and thereby could further increase treatment responses. We, therefore, pre-treated cells with marizomib for 24 h, followed by the addition of IZI1551 and monitoring of cell death. In comparison to co-stimulation, the pre-treatment with marizomib triggered earlier responses and substantially enhanced cell death, as observed by PI positivity in populations of N160125 and GTCC9 cells (Fig. 3C**;** Supplemental Fig. 2B). Determining apoptosis flow cytometrically provided comparable results, with marizomib pre-treatment significantly accelerating and/or enhancing cell death (Supplemental Fig. 2C). Enhanced apoptosis induction upon pre-treatment also manifested when lowering both drugs concentrations (100 pM of IZI1551, 40 nM of marizomib) (Fig. 3D), demonstrating that time-shifted exposure allows lowering the concentrations of both drugs, which in a translational setting could be desirable, and that the time-shifted addition is still more effective than simultaneous drug application.

**Fig. 4:**
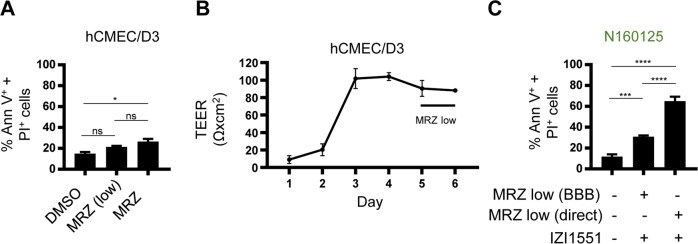
Marizomib sufficiently penetrated a human BBB model to sensitize GBM cells to IZI1551-induced cell death. **A** Human BBB cells hCMEC/D3 were tested for their responsiveness to marizomib (40 nM (low); 80 nM) by Annexin V/PI-based flow cytometry. Data represent mean ± SEM from three independent experiments. *p ≤ 0.05; ns, not significant; one-way ANOVA followed by Tukey post hoc test. **B** TEER was measured for 5 days following 24 h of growth of hCMEC/D3 on culture inserts. On the 5th day, 40 nM marizomib was added and the TEER was monitored for an additional 24 h. Data represent mean ± range of *n* = 2 measurements. **C** Cell death in N160125 cells measured by Annexin V/PI-based flow cytometry. Where indicated, cells were pre-treated with marizomib (40 nM) directly or with medium from the basolateral side of an hCMEC/D3 BBB (40 nM marizomib on the apical side for 24 h). Data represent mean ± SD of three technical replicates ***p ≤ 0.001; ****p ≤ 0.0001; one-way ANOVA followed by Tukey post hoc test.

Marizomib was described as the so far only BBB-permeant proteasome inhibitor [10, 11] so that systemic marizomib pre-treatments could potentially be used to prime GBM cells for TRAIL receptor-induced apoptosis. We therefore next studied if the amounts of marizomib that can cross the BBB are sufficient to confer sensitization to IZI1551. To this end, we used an in vitro BBB model grown from human brain endothelial cells (hCMEC/D3). BBB cells were largely resistant to marizomib (Fig. 4A) and formed a tight BBB, as characterized by high transepithelial/transendothelial electrical resistance (TEER) (Fig. 4B). Importantly, marizomib did not compromise the TEER of a fully formed barrier (Fig. 4B). Following exposure of the BBB to marizomib, medium from the apical or basolateral sides was added to GBM cells, followed by the addition of IZI1551. While the presence of the BBB reduced overall treatment responsiveness to the combination of low marizomib concentrations and IZI1551 in the pre-treatment scenario, cell death could still clearly be detected in this setting (Fig. 4C). Overall, these results demonstrate that marizomib pre-treatment can further enhance IZI1551 responsiveness and that such sensitization effects can also be observed in conditions where marizomib needs to cross a simple model of a human BBB.

### Non-responders can be sensitized to IZI1551-induced cell death by Mcl-1 antagonism

**Fig. 5:**
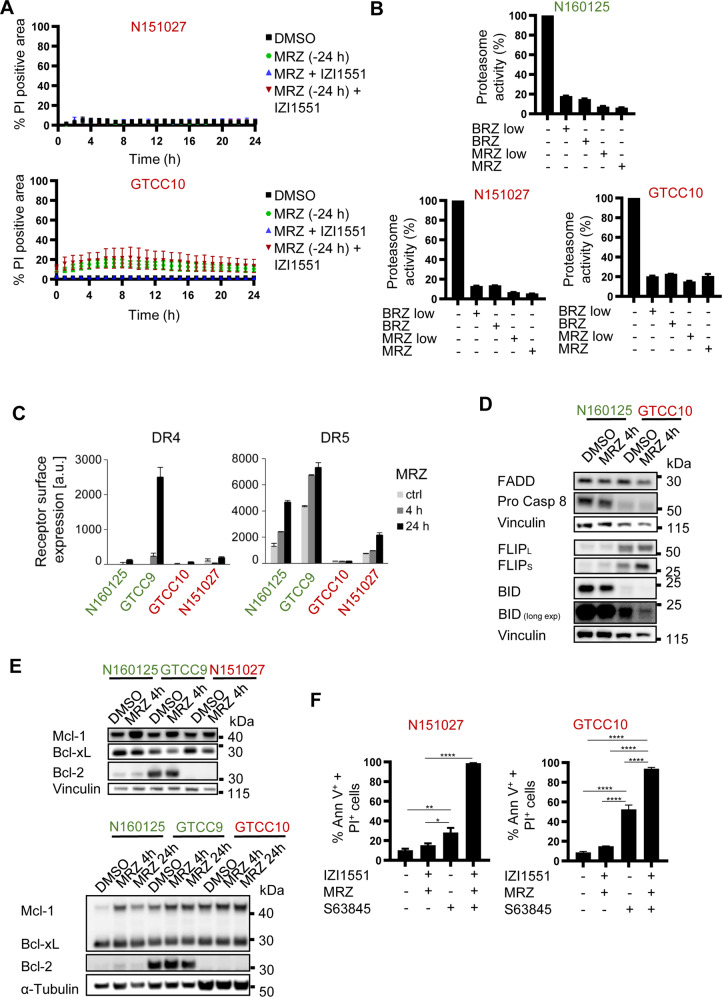
Non-responders can be sensitized to IZI1551-induced cell death by Mcl-1 antagonism. **A** Quantification of cell death kinetics, calculated as a percentage of PI-positive cell areas. Cells were co-treated with IZI1551 (1 nM) and marizomib (80 nM) simultaneously or pre-treated with marizomib for 24 h (MRZ −24 h) before the addition of IZI1551. Representative results from one out of 3 independent experiments are shown. Error bars represent the SD of 3 technical replicates. **B** Marizomib inhibited proteasome activities both in responder and non-responder cell lines. Cells were treated with 40 nM (low) or 80 nM of marizomib or bortezomib for 4 h and CT-L activities of the proteasomes were measured from total cell lysates by cleavage of Suc-LLVY-AMC. Bars indicate % of activity related to untreated controls. Data are shown as mean ± range of *n* = 2 measurements. **C** Surface expression of death receptors DR4 and DR5 in responsive and resistant cell lines. Cells were treated with marizomib (80 nM) and surface amounts were determined by flow cytometric measurements. Data show mean plus range. Similar results were obtained in independent repeat experiments. **D** Procaspase-8; FADD; FLIP and BID proteins are detected at different expression levels in responsive and resistant cells. Cells were treated with marizomib and the indicated proteins were detected in whole-cell lysates. Vinculin served as a loading control. Similar results were obtained in independent repeat experiments. **E** Anti-apoptotic Bcl-2 family proteins were detected in different cell lines. Cells were treated with marizomib (80 nM) and the indicated proteins were detected in whole-cell lysates. Vinculin or α-Tubulin served as loading controls. Similar results were obtained in independent repeat experiments. **F** Annexin V/PI-based flow cytometry of cells treated with 10 μM of S63845; 1 nM IZI1551 plus 80 nM MRZ or the combination thereof for 24 h. Data represent mean ± SEM of three independent experiments. *p ≤ 0.05; **p ≤ 0.01; ****p ≤ 0.0001; one-way ANOVA followed by Tukey post hoc test.

Next, we tested if marizomib pre-treatment would also sensitize non-responder cell lines to IZI1551. Kinetic and quantitative analyses in N151027 and GTCC10 cells, however, demonstrated that these cells remained resistant to treatment despite 24 h pre-treatment with marizomib (Fig. 5A, Supplemental Fig. 3A). Since marizomib efficiently inhibited proteasomes in responder and non-responder cells (Fig. 5B), the reason for poor responsiveness might lie downstream within the apoptosis signaling networks or within the IZI1551-induced apoptosis initiation phase. We, therefore, studied if the expression of key proteins involved in initiating TRAIL receptor-dependent apoptosis differed between responder and non-responder cell lines and if such differences could possibly explain IZI1551 resistance.

At the level of death receptor expression, differences explaining responsiveness or resistance could not be identified when analyzing total cellular receptor expression. The non-responder cell line GTCC10 expressed low amounts of DR5 and undetectable amounts of DR4 (Supplemental Fig. 3B). However, this was also the case for the responder cell line N160125 (Supplemental Fig. 3B). Similar results were also obtained for the non-responder cell line N151027 when compared to responder cells (Supplemental Fig. 3B). However, when analyzing cell surface amounts of DR4 and DR5, non-responder cell lines tended to present with lower receptor amounts and failed to accumulate additional DR4/DR5 amounts in response to proteasome inhibition (Fig. 5C). We then also compared the expression of FADD, procaspase-8, and FLIP variants as components of the DISC, as well as the expression of the caspase-8 substrate BID between responder and non-responder cell lines. While FADD levels were comparable in responsive and resistant cells, both non-responder cell lines showed lower procaspase-8 and higher FLIP expression, with GTCC10 cells also expressing low amounts of BID (Fig. 5D, Supplemental Fig. 3C). Marizomib treatment did not notably change baseline expression amounts, with the exception of the anti-apoptotic protein FLIP, which increased (Fig. 5C, D, Supplemental Fig. 3B, C). Overall, the protein expression signatures in non-responders cells at baseline, as well as after marizomib treatment, therefore, appear associated with a low competency to induce extrinsic apoptosis. Alterations in protein expression amounts between 2D and 3D conditions indicated increases in Bcl-2 amounts, while the amounts of other anti-apoptotic proteins dropped (Supplemental Fig. 3D). Since response profiles were largely maintained between 2D and 3D scenarios (Figs. 1, 2), such alterations in protein expression apparently largely failed to broadly affect treatment susceptibility.

Since the early signaling steps towards apoptosis initiation in response to marizomib/IZI1551 are likely insufficiently strong to trigger the self-amplifying apoptosis execution phase, we next sought to identify if sensitization to the mitochondrial apoptosis pathway downstream of caspase-8 activation could serve as a strategy that restores apoptosis competency in non-responder cell lines. The threshold for mitochondrial apoptosis is co-determined by the expression of anti-apoptotic Bcl-2 protein family members, such as Mcl-1, Bcl-xL, and Bcl-2, and targeted therapeutics for all three family members have been developed [32, 33]. Interestingly, Bcl-2 expression in non-responder cell lines was undetectable, whereas Mcl-1 and Bcl-xL both were expressed (Fig. 5E). Indeed, Bcl-2 antagonist ABT-199 failed to enhance responsiveness in GTCC10 cells, in line with the lack of Bcl-2 expression in non-responders (Supplemental Fig. 3E). As it also seemed that Mcl-1, a high turnover protein, tended to accumulate after proteasome inhibition, we tested if antagonizing Mcl-1 by S63845 could restore apoptosis susceptibility to marizomib/IZI1551 combination treatment. Indeed, both non-responder cell lines responded with apoptosis in this setting (Fig. 5F), as did a 3D spheroid culture model (Supplemental Fig. 3F).

Taken together, these findings demonstrate the effectiveness of lowering the mitochondrial apoptosis threshold to allow apoptosis execution upon marizomib/IZI1551 treatment in otherwise resistant GBM cells.

## Discussion

In this study, we analyzed the responsiveness of low passage primary GBM cell lines to the combination of the BBB-permeant proteasome inhibitor marizomib and a latest-generation TRAIL receptor agonist. We found that the majority of cell lines responded synergistically to combination treatments, both in 2D and 3D spheroid cultures. In cases of treatment resistance, lowering the mitochondrial apoptosis threshold appeared sufficient to restore apoptosis sensitivity.

Proteasome inhibition induces complex cellular stress responses that, if stress remains unresolved, ultimately result in cell death. Cell death upon proteasome inhibition most prominently induces intrinsic apoptosis [34, 35], whereas death ligands primarily induce extrinsic apoptosis as the main cell death modality [36]. However, besides inducing apoptosis, death ligands can also induce necroptosis, as long as caspase-8 activation remains compromised and the kinase signaling cascade consisting of RIPK1, RIPK3, and MLKL can be activated [36, 37]. For the combination of marizomib/IZI1551, however, we observed that caspase inhibition was sufficient to entirely prevent cell death, suggesting that alternative cell death mechanisms such as necroptosis remain irrelevant in this setting. While necroptosis competency has been reported in glioma, such cases might be rare, since RIPK3 expression is frequently epigenetically silenced [38–40]. Furthermore, proteasome inhibition suppresses necroptosis competency, as shown in TNFα/zVAD-fmk/IAP antagonist treated macrophages and HT29 colon cancer cells [41, 42].

The potency of marizomib to sensitize to a 2nd generation TRAIL receptor agonist has not been evaluated yet for GBM or other cancer models. However, it was shown that marizomib potentiates cell death induction upon activation of TNF-receptors by TNFα in leukemia and multiple myeloma cells [43]. Similarly, proteasome inhibitors such as MG132 and bortezomib can enhance apoptosis induced by recombinant human TRAIL [18–20]. Overall, this indicates that the complex mechanisms giving rise to response synergies between proteasome inhibitors and death receptor ligands are likely identical across all death ligands and proteasome inhibitors, with the added benefit that marizomib is BBB permeant. Among the sensitization mechanisms, however, one aspect might differ between models of glioma and other cancers. The stabilization of IκBα by proteasome inhibition is often referred to as a mechanism by which pro-survival NFκB signaling branches that can be triggered by death receptor activation are blocked, yet NFκB signaling surprisingly might have a pro-death function specifically in the glioma setting [44].

Our work demonstrates that pre-treatment with marizomib strongly enhances GBM cell responsiveness to IZI1551. The relative timing of drug exposure and thereby the optimization of treatment responsiveness typically is not systematically studied in cell biological studies, even though the need to consider the sequence of drug additions in the overall strategy of inducing cell death in cancer cells has been flagged before and the influence of treatment schedules and sequences on synergisms are known since a long time [45, 46]. However, modifying relative timings of drug exposures by pre-treatment and post-treatment in clinical trials typically cannot reasonably be integrated into trial designs, in particular in rare cancers such as GBM. Pre-treatment strategies thus naturally must be driven by pre-clinical experimental evidence and knowledge, which in our cases orientated along marizomib concentrations that are translationally relevant [10, 47]. In the case of marizomib combination treatments with anti-cancer biologics such as TRAIL receptor agonists, this indeed could be accommodated clinically by systemic marizomib therapy prior to surgical removal of the bulk tumor mass and implantation of carriers that release large bio-molecules within the tumor and adjacent brain areas. Implants such as carmustine wafers have been tested clinically already and technologies to expand such strategies to biologics, for example, based on injectable or implantable hydrogels, are in development [48–50]. It would likewise be interesting to study in the future how and if this treatment combination could be combined with standard of care radio-chemotherapy or if it rather would take a position as salvage therapy. Toxicities upon marizomib treatment so far appear manageable in combination with temozolomide-based radio-chemotherapy [51], so that additional combinations could be considered.

## Materials and methods

### Reagents and antibodies

Q-VD-Oph was purchased from Selleckchem (Houston, TX, USA). Marizomib was purchased from Sigma-Aldrich (Munich, Germany). S63845 was purchased from APExBIO Technology (Houston, TX, USA). IZI1551 (TRAIL) and Annexin V-EGFP were produced in-house. DMSO was purchased from Carl Roth (Karlsruhe, Germany). Propidium Iodide was purchased from Sigma-Aldrich (Munich, Germany). Digitonin was purchased from SERVA Electrophoresis (Heidelberg, Germany). The following antibodies were used for western blotting: rabbit monoclonal Caspase-8 (#4790, clone D35G2), mouse monoclonal Caspase-8 (#9746, clone 1C12), rabbit polyclonal Caspase-3 (#9662), mouse monoclonal α-Tubulin (#3873, clone DM1A), mouse monoclonal GAPDH (#97166, clone D4C6R), rabbit monoclonal DR4 (#42533S, clone D9S1R), rabbit monoclonal DR5 (#8074, clone D4E9) XP, rabbit polyclonal DR5 (#3696S), rabbit polyclonal Bcl-2 (#2872), rabbit monoclonal Mcl-1 (#94296, clone D2WE9), rabbit monoclonal Bcl-xL (#2764S, clone 54H6), rabbit monoclonal FLIP (#56343, clone D5J1E), rabbit monoclonal FLIP (#8510S, clone D16A8), rabbit polyclonal BID (#2002), rabbit polyclonal FADD (#2782), rabbit polyclonal β-Actin (#4967), all purchased from Cell Signaling Technologies (CST, Danvers, MA, USA). Mouse monoclonal PARP (#556494, clone 4C10-5), mouse monoclonal Caspase-8 (#556466, clone B9-2), mouse monoclonal FADD (#610400, clone 1), mouse monoclonal BID (#611528, clone 7), and mouse monoclonal XIAP (#610716, clone 28/hILP) antibodies were purchased from BD Bioscience (Heidelberg, Germany). Mouse monoclonal Vinculin (#sc-73614, clone 7F9) and mouse monoclonal Bcl-2 (#sc-509, clone 100) antibodies were purchased from Santa Cruz Biotechnology (SCBT, Heidelberg, Germany). Rabbit monoclonal GAPDH (#MAB374, clone 6C5) antibody was purchased from Merck Millipore (Darmstadt, Germany). Anti-mouse IgG HRP-linked antibody (#115-035-062) and anti-rabbit IgG HRP-linked antibody (#111-035-144) were purchased from Dianova (Dianova GmbH, Hamburg, Germany). The following antibodies were used for flow cytometry: mouse monoclonal TRAILR1 (#MAB347, clone 69036) and mouse monoclonal TRAILR2 (#MAB6311, clone 71908) purchased from R&D Systems (Wiesbaden-Nordenstadt, Germany); purified mouse IgG1,κ isotype control (#554121) and purified mouse IgG2b,κ isotype control (#555740) purchased from BD Biosciences (Heidelberg, Germany); goat anti-mouse Alexa 488 (IgG (H + L) highly cross-adsorbed, A-11029) purchased from Thermo Fisher Scientific (Waltham, MA, USA).

### Cell culture

Primary cell lines were derived from primary GBM tumors (N160240, N160125, N150385, and N150661; generated at ICM Paris) or recurrent GBM tumors (GTCC9 and GTCC10; generated at ECM Rotterdam). GBM tissue samples were provided by the neuropathology laboratory of Pitie-Salpetriere University Hospital (Paris, France) or the Department of Neurosurgery of the ErasmusMC (Rotterdam, The Netherlands), and obtained as part of routine resections from patients under their informed consent (ethical approval numbers AC-2013-1962, MEC-2013-090 under the auspices of the ethics committees of the aforementioned institutions). Cells were utilized only until passage number 25 and were grown in neurosphere medium (NS) freshly prepared once every 2 weeks as follows: Dulbecco’s Modified Eagle Medium (Nutrient Mixture F-12-DMEM/F-12, Thermo Fisher Scientific, Gibco, Waltham, MA, USA) supplemented with 2% B27 (Thermo Fisher Scientific, Gibco, Waltham, MA, USA); 20 ng/ml bFGF (Peprotech, Hamburg, Germany); 20 ng/ml EGF (Peprotech, Hamburg, Germany); 5 μg/ml Heparin (Alfa Aesar, Ward Hill, MA, USA) and 1% Pen/Strep. Cell dissociation was performed using Accutase (ThermoFisher Scientific, MA, USA). For 2D cultures, cells were seeded on plates coated with 1:100 Cultrex® Reduced Growth Factor Basement Membrane Matrix (BME, Trevigen, Wiesbaden-Nordenstadt, Germany). For the generation of spheroids, cells were plated onto cultureware pre-rinsed with Anti-Adherence Rinsing Solution (STEMCELL Technologies, Cologne, Germany). Blood–brain barrier hCMEC/D3 endothelial cells were purchased from Merck Millipore, (Darmstadt, Germany) and cultured in Endothelial Cell Growth Basal Medium MV 2 (C-22221, Promo cell GmbH, Heidelberg, Germany) with supplements including 0.05 ml/ml of Fetal Calf Serum, 0.004 ml/ml of Endothelial Cell Growth Supplement, 10 ng/ml of Epidermal Growth Factor, 90 μg/ml of Heparin, 1 μg/ml of Hydrocortisone and 1 ng/ml of basic Fibroblast Growth Factor (C-39221, Promo cell GmbH, Heidelberg, Germany) on Gibco™ Collagen type I, Rat Tail-coated flasks (10 μg/cm^2^, Thermo Fisher Scientific, Gibco, Waltham, MA, USA) until passage 10. All cell lines were tested for mycoplasma infection.

### Viability assay

Cell viability was assessed using WST-1 cell proliferation reagent (ThermoFisher Scientific, MA, USA) according to the manufacturer’s instructions. Briefly, 3000 cells/well were plated in a 96 well plate 24 h prior to the experiments. Twenty-four hours after treatment, 1:10 WST-1 reagent was added. Following 3 h of incubation, plates were read by spectrophotometry at 450 and 620 nm wavelength. Reads at 620 nm (background signal) were subtracted from reads at 450 nm. Values were normalized to untreated controls.

If needed, the cell viability of spheroids was optically assessed via staining with the cell-permeant fluorescent probe Calcein AM (ThermoFisher Scientific, MA, USA). Spheroids were resuspended in DMEM/F-12 medium containing 4 μM of equilibrated Calcein AM. After 30 min of incubation at 37 °C and 5% CO_2_, spheroids were imaged by fluorescence microscopy for morphological documentation.

### Flow cytometry

Cells were stained with Propidium Iodide and Annexin V-EGFP in Annexin V binding buffer (BD Biosciences, Heidelberg, Germany) for 5 min at room temperature. Measurements were performed on a MACSQuant instrument (Miltenyi Biotec, Bergisch Gladbach, Germany) or a BD LSRII SORP HTS cytometer (BD Biosciences, NY, USA), and data were processed with Flowing software ((Turku Centre for Biotechnology, Finland)) or MACSQuantify^TM^ (Militenyi Biotec, Bergisch Gladbach, Germany).

### Time-lapse imaging

Twenty-four hours prior to experiments, cells were seeded into 96-well plates coated with 1:100 BME. Cells were then treated as indicated and the plates were placed into an IncuCyte S3 Live Cell Analysis System (Essen BioScience). Confluency and the percentage of PI-positive areas in 2 fields of view per well in 3 wells per treatment were quantified using the semi-automated IncuCyte S3 software (Essen BioScience). Values were normalized to the percentage of PI-positive area upon digitonin treatment.

### Long term survival assay

Following 24 h of treatment, triplicates of 500 survivor cells per condition each were re-seeded in 96 well plates coated with 1:100 BME. Six days later, cells were imaged at ×10 magnification using an EVOS M5000 microscope (ThermoFisher Scientific Waltham, MA, USA), and their viability was assessed with a WST-1 assay. Cells were counted in an exact volume of 100 μL per condition using a MACSQuant flow cytometer and proliferation capacity was normalized by relating the numbers of cells to untreated controls.

### Western blotting

2D-cultured or 3D-cultured cells were washed with ice-cold PBS before they were lysed in lysis buffer (150 mM NaCl; 20 mM TRIS; 1 mM EDTA; 1% (v/v) Triton x-100, pH 7,6) with 1:25 Complete protease inhibitors (Roche) for 15 min on ice. Cellular debris was removed by centrifugation at 16,000 *g* for 15 min at 4 °C. Protein concentrations were quantified by Bradford assay. Equal amounts of proteins were supplemented with 5× Laemmli sample buffer (10% SDS, 312.5 mM Tris pH 6.8, 25% β-mercaptoethanol, 25% glycerin, 0.05% bromphenol blue; all chemicals were purchased from Carl Roth, Karlsruhe, Germany) and heated to 95 °C for 5 min. Proteins were separated on Nu-Page 4–12% Bis-Tris gels (Invitrogen, Carlsbad, CA, USA) and transferred to nitrocellulose membranes using an iBlot 2 gel transfer device (Invitrogen, Carlsbad, CA, USA). After 1 h blocking with blocking reagent (Roche Diagnostics, Mannheim, Germany) diluted in TBST (1%), the membranes were incubated with primary antibodies (diluted in TBST with 0.5% blocking reagent) overnight. After washing with TBST, membranes were incubated with an HRP-coupled secondary antibody (diluted in TBST with 0.5% blocking reagent) for 1 h at room temperature. Following three further washing steps, proteins were detected by incubating the membranes with an HRP substrate (Thermo Scientific Pierce Protein Biology, Waltham, MA, USA) and detecting the signals with an ECL imager (Amersham Imager 600, GE Healthcare, Freiburg, Germany GmbH).

### hCMEC/D3 BBB model

hCMEC/D3 cells were grown on PET 6-well ThinCert™ Cell Culture Inserts with a 0.4 μm pore size (Greiner Bio-One, Frickenhausen, Germany) coated with Gibco™ Collagen type I, Rat Tail-coated flasks (10 μg/cm^2^, Thermo Fisher Scientific, Gibco, Waltham, MA, USA) at a density of 0.4 × 10^6^ cells/well and allowed to reach confluence for another five days. Experiments were performed when the transendothelial electrical resistance (TEER) exceeded 80 Ω × cm^2^. TEER was measured every day by an EVOM voltohmmeter (World Precision Instruments, Sarasota, FL, USA) combined with STX-2 electrodes. Recorded resistance was related to the surface area of the transwell insert (Ω × cm^2^). Resistance of cell-free inserts was subtracted from the measured data.

### Proteasome activity assay

Chymotrypsin-like proteasome activity was measured using a fluorigenic peptide N-succinyl-Leu-Leu-Val-Tyr-7-amino-4-methyl-coumarin (suc-LLVY-AMC) (Merck Millipore/Calbiochem, Darmstadt, Germany). Cells were treated with proteasome inhibitors for 4 h and were lysed using lysis buffer (10 mM HEPES, 42 mM KCl, 5 mM MgCl_2_, 0.1 mM EDTA, 0.1 mM EGTA, 1 mM DTT, 0.5% (w/v) CHAPS). Lysates were incubated with reaction buffer (25 mM HEPES, pH 7.4, 0.5 mM EDTA, pH 8) containing 20 μM suc-LLVY-AMC. Using 380 nm excitation, fluorescence was measured at 460 nm and 37 °C using a plate reader with the appropriate filters (Spark^®^, Tecan, Männedorf, Switzerland). Fluorescence signals were normalized to the protein concentrations, which were determined by Bradford assay and related to untreated, autofluorescence-corrected controls (100% activity).

### Flow cytometric analysis of death receptor surface expression

For measurements of cell surface receptor amounts, cells were suspended in cold PBA (1.5 × 10^4^ cells per sample, PBS +0.25% (w/v) bovine serum albumin (BSA, Sigma-Aldrich, Munich, Germany) + 0.02% (w/v) NaN_3_ (Carl Roth, Karlsruhe, Germany) in ddH_2_0) containing primary antibody or isotype control (4 µg/ml/sample, on ice). After 30 min incubation, cells were washed with PBA and resuspended in PBA containing secondary antibody (10 µg/ml on ice, 45 min in the dark). Thereafter, cells were washed with PBA, and measurements performed on a MACSQuant instrument. All flow cytometric data were analyzed by MACSQuantify (MACS Miltenyi Biotec, Bergisch Gladbach, Germany).

### Statistical analysis

Statistical analysis was performed using GraphPad Prism 9 (GraphPad Software, San Diego, CA, USA). Data are shown as mean values plus and minus the standard deviation (SD) or standard error of the mean (SEM) unless stated otherwise in the figure legends. Statistical significance of differences between groups was verified using the stated significance tests. Significance level were denoted with asterisks: *p ≤ 0.05; **p ≤ 0.01; ***p ≤ 0.001; ****p ≤ 0.0001. Synergy scores were calculated by Webb’s fractional product as previously reported [24], scores of <0.9 were considered as synergistic. Unless otherwise stated, data are from three independent repeat experiments.

## Supplementary information

Supplemental Figs Legends

Fig S1

Fig S2

Fig S3
