# Supplementary material for: Marizomib sensitizes primary glioma cells to apoptosis induced by a latest-generation TRAIL receptor agonist
Source: Cell Death Dis. 2021 Jun 24;12(7):647. doi: 10.1038/s41419-021-03927-x (PMC8225658; doi:10.1038/s41419-021-03927-x)
Supplement: Supplementary file 1 — Supplemental Figs Legends [file 41419_2021_3927_MOESM1_ESM.docx]

**Supplemental Figure Legends**

**Supplemental Figure 1:**

1. Comparison between cell viability and cell death measurements. Cell viability was assessed by WST-1 cell proliferation assay. Cell death was assessed via flow cytometry of cells stained with both Annexin V and PI. n = 3; SEM < 10%.
2. Representative micrographs of N160125 spheroids stained with Calcein AM (green; live cells) following 24 h of exposure to 1 nM of IZI1551 and 80 nM of marizomib.

**Supplemental Figure 2:**

1. Cells were treated with IZI1551 (1 nM), marizomib (80 nM) or a combination of both in presence of propidium iodide and imaged at 0 h and 4 h. Micrographs are representative of 3 independent experiments.
2. Following pre-treatment with marizomib for 24 h, cells were treated with IZI1551 and imaged as in (A). Micrographs are representative of 3 independent experiments are shown.

**(C)** Annexin V/PI-based flow cytometry of cells co-treated with IZI1551 (1 nM) and MRZ (80 nM) simultaneously or pre-treated with MRZ for 24 h (MRZ -24 h) before the addition of IZI1551. Data represent mean ± SEM from three independent experiments. ** = p ≤ 0.01; *** = p ≤ 0.001 ****. ns, not significant (two-way ANOVA followed by Tukey or Sidak post hoc test).

**Supplemental Figure 3:**

1. Time lapse monitoring of cell death. Cells were co-treated with IZI1551 (1 nM) and marizomib (80 nM) simultaneously or pre-treated with marizomib for 24 h (MRZ -24 h) before the addition of IZI1551 and then imaged for 24 h in presence of propidium iodide. Micrographs representative of 3 independent experiments are shown.

**(B)** Death receptors DR4 and DR5 can be detected both in responsive and resistant cell lines. Cells were treated for 4 h or 24 h with marizomib (80 nM). 15 μg of whole-cell lysates were analysed for the indicated proteins by western blotting. α-Tubulin or Vinculin served as loading control. Similar results were obtained in independent repeat experiments.

**(C)** Procaspase-8; FADD; FLIP and BID proteins are detected at different expression levels in responsive and resistant cells. Cells were treated as in (B) and the indicated proteins were detected from 15 μg of whole-cell lysates. Vinculin served as loading control. Similar results were obtained in independent repeat experiments.

**(D)** Representative immunoblots showing expression amounts of apoptosis regulators in 2D and 3D culturing conditions. GAPDH served as loading control.

**(E)** Annexin V/PI-based flow cytometry of cells treated with 10 μM of ABT-199; 1 nM IZI1551 plus 80 nM MRZ or the combination thereof for 24 h. Data represent mean ± SD of triplicate samples.

**(F)** Annexin V/PI-based flow cytometry of N151027 spheroids treated with 10 μM of S63845; 1 nM IZI1551 plus 80 nM MRZ or the combination thereof for 24 h. Data represent mean ± SEM of three independent experiments. ** = p ≤ 0.01; one-way ANOVA followed by Tukey post hoc test.
