# Supplementary figures and images for: Marizomib sensitizes primary glioma cells to apoptosis induced by a latest-generation TRAIL receptor agonist
Source: Cell Death Dis. 2021 Jun 24;12(7):647. doi: 10.1038/s41419-021-03927-x (PMC8225658; doi:10.1038/s41419-021-03927-x)

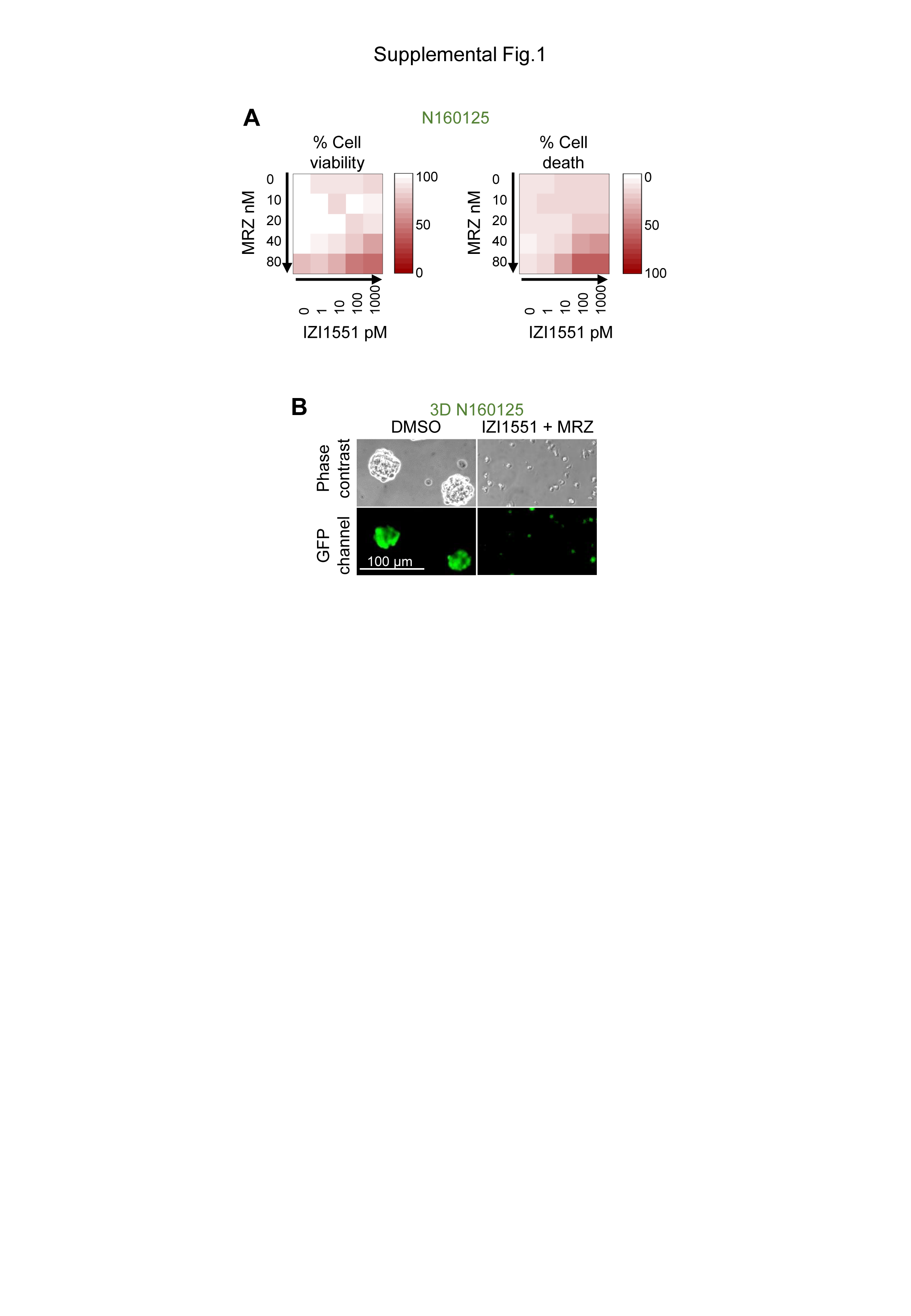

Supplement: Supplementary file 2 — Fig S1 [file 41419_2021_3927_MOESM2_ESM.tif]

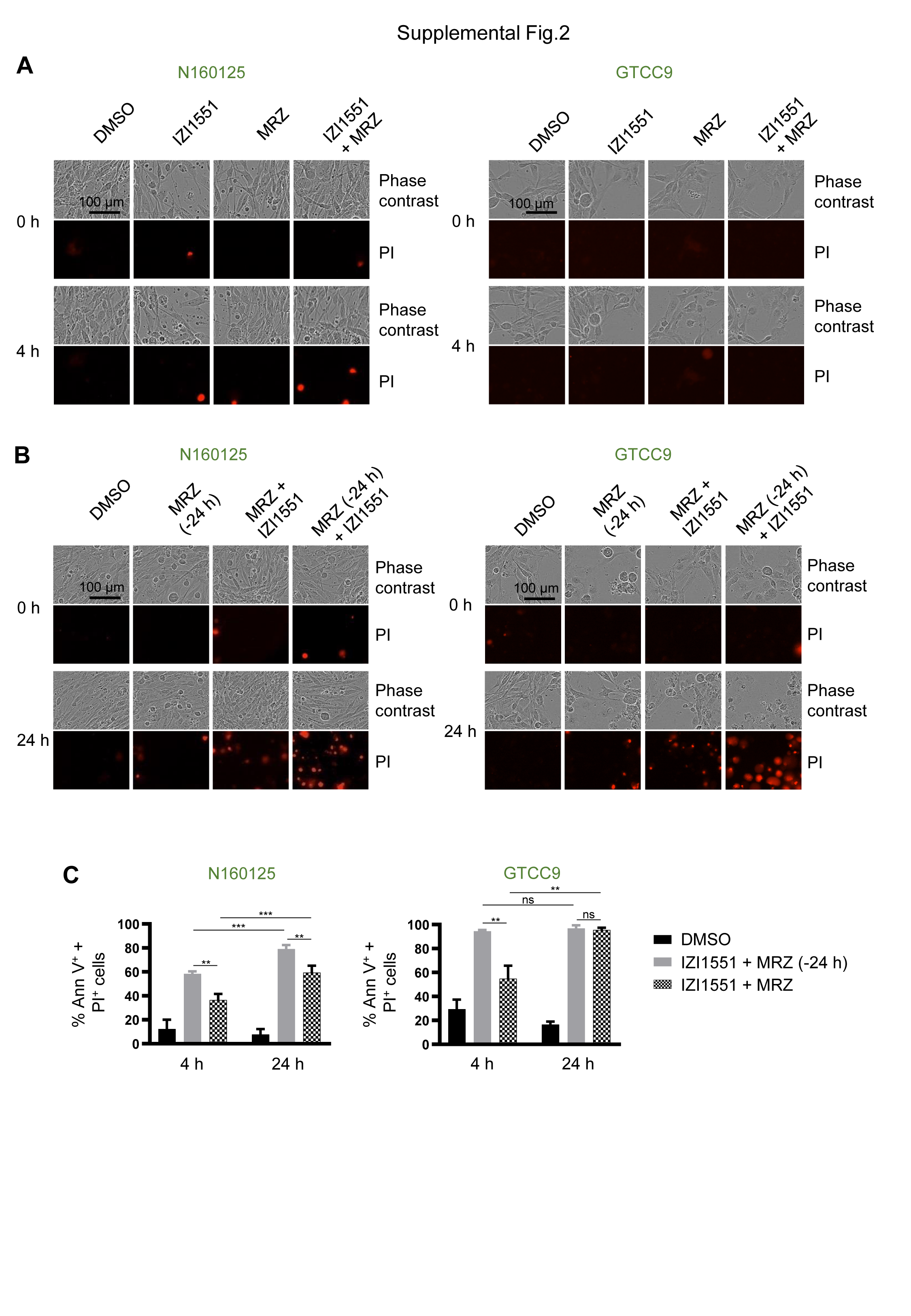

Supplement: Supplementary file 3 — Fig S2 [file 41419_2021_3927_MOESM3_ESM.tif]

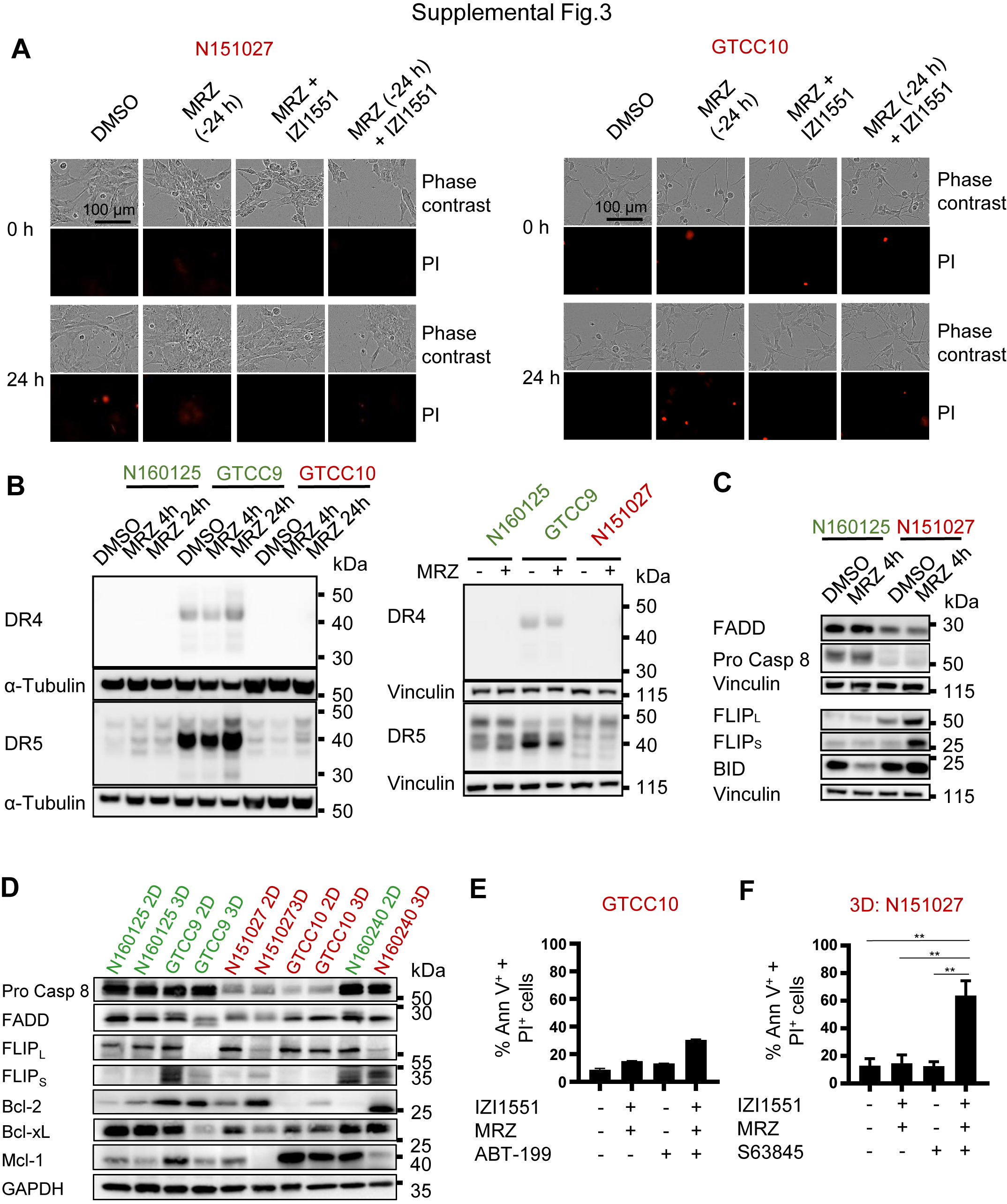

Supplement: Supplementary file 4 — Fig S3 [file 41419_2021_3927_MOESM4_ESM.tif]
